# Impact of levosimendan on weaning from peripheral venoarterial extracorporeal membrane oxygenation in intensive care unit

**DOI:** 10.1186/s13613-019-0503-1

**Published:** 2019-02-01

**Authors:** Shamir Vally, Cyril Ferdynus, Romain Persichini, Bruno Bouchet, Eric Braunberger, Hugo Lo Pinto, Olivier Martinet, David Vandroux, Thomas Aujoulat, Jérôme Allyn, Nicolas Allou

**Affiliations:** 1 Réanimation polyvalente, Centre Hospitalier Universitaire Félix Guyon, 97405 Saint-Denis, Allée des Topazes, France; 2 Unité de Soutien Méthodologique, CHU de La Réunion, Saint-Denis, France; 3 INSERM, CIC 1410, Saint-Pierre, France; 4 Chirurgie cardiaque, Centre Hospitalier Universitaire Félix Guyon, 97405 Saint-Denis, Allée des Topazes, France; 5 Réanimation polyvalente, Hôpital Félix Guyon, Bellepierre, 97405 Saint-Denis, France

**Keywords:** Extracorporeal membrane oxygenation, Levosimendan, Weaning, Mortality

## Abstract

**Background:**

Few data are available on the impact of levosimendan in refractory cardiogenic shock patients undergoing peripheral venoarterial extracorporeal membrane oxygenation (VA-ECMO). The aim of this study was to evaluate the impact of levosimendan on VA-ECMO weaning in patients hospitalized in intensive care unit (ICU).

**Methods:**

This retrospective cohort study was conducted in a French university hospital from 2010 to 2017. All patients hospitalized in ICU undergoing VA-ECMO were consecutively evaluated.

**Results:**

A total of 150 patients undergoing VA-ECMO were eligible for the study. Thirty-eight propensity-matched patients were evaluated in the levosimendan group and 65 in the non-levosimendan group. In patients treated with levosimendan, left ventricular ejection fraction had increased from 21.5 ± 9.1% to 30.7 ± 13.5% (*P* < 0.0001) and aortic velocity–time integral from 8.9 ± 4 cm to 12.5 ± 3.8 cm (*P* = 0.002) 24 h after drug infusion. After propensity score matching, levosimendan was the only factor associated with a significant reduction in VA-ECMO weaning failure rates (hazard ratio = 0.16; 95% confidence interval 0.04–0.7; *P* = 0.01). Kaplan–Meier survival curves showed that survival rates at 30 days were 78.4% for the levosimendan group and 49.5% for the non-levosimendan group (*P* = 0.02). After propensity score matching analysis, the difference in 30-day mortality between the two groups was not significant (hazard ratio = 0.55; 95% confidence interval 0.27–1.10; *P* = 0.09).

**Conclusions:**

Our results suggest that levosimendan was associated with a beneficial effect on VA-ECMO weaning in ICU patients.

## Introduction

Venoarterial extracorporeal membrane oxygenation (VA-ECMO) is increasingly being used as a support system for patients with cardiogenic shock refractory to conventional medical therapies [1]. Levosimendan is a calcium-sensitizing inotropic agent that improves myocardial function in patients with cardiogenic shock [2]. Unlike other inotropes like dobutamine, levosimendan has anti-inflammatory properties and does not increase myocardial oxygen consumption [3]. Nevertheless, debates continue in clinical practice regarding the beneficial effects of levosimendan in patients with cardiogenic shock or low cardiac output syndrome who are not treated with VA-ECMO [4–7]. While levosimendan has been shown to improve endothelial function and to increase cardiac index in cardiogenic shock patients undergoing VA-ECMO [8], few data are available on the impact of levosimendan in refractory cardiogenic shock patients undergoing VA-ECMO [9–11]. One study has suggested that levosimendan has beneficial effects on survival and VA-ECMO weaning, but only after cardiac surgery [10]. It may be that levosimendan should be administered only to specific cardiogenic shock patients, in particular those undergoing VA-ECMO. The aim of this study was to evaluate the impact of levosimendan on VA-ECMO weaning in patients hospitalized in intensive care unit (ICU).

## Materials and methods

This study was approved by the Institutional Review Board of the Ethics Committee of the French Intensive Care Society (CE SRLF 18-03) and was declared to the Commission nationale de l’informatique et des libertés (CNIL MR-003, N° 2000694). The need for informed consent was waived because of the observational and retrospective nature of the study.

### Selection of the study sample

This retrospective cohort study was conducted between January 2010 and March 2017 in the 23-bed mixed medical/surgical ICU of a French university hospital.

All patients hospitalized in ICU undergoing VA-ECMO were consecutively evaluated. Exclusion criteria were: age < 18 years, VA-ECMO duration < 2 days and central VA-ECMO treatment.

During the study period, levosimendan and other catecholamines were administered at the physician’s discretion to patients undergoing VA-ECMO. Levosimendan (12.5 mg diluted in 100 mL of NaCl 0.9%) was administered without bolus as a continuous infusion at a dose of 0.2 μg per kilogram per minute during 24 h.

For patients with mean arterial pressure above 65 mmHg with pulsatile flow, VA-ECMO weaning was considered daily and VA-ECMO flow was gradually decreased to a minimum of 1–1.5 L/min. VA-ECMO was removed in patients meeting the following criteria: mean arterial pressure > 65 mmHg; low doses of administered catecholamine (norepinephrine < 0.1 µg/kg/min, dobutamine < 5 µg/kg/min and no epinephrine); PaO_2_/FiO_2_ ratio > 100 mmHg; left ventricular ejection fraction > 20%; and aortic velocity–time integral > 12 cm [12, 13].

### Data collection

Data were collected on: age; gender; Simplified Acute Physiology Score 2; body mass index > 30 kg/m^2^; previous coronary artery disease; hypertension; chronic renal failure with dialysis; chronic obstructive pulmonary disease; diabetes mellitus; history of congestive heart failure; immunosuppression (immunosuppressive disease, hematologic disease, treatment with immunosuppressive drugs within the previous 30 days, corticosteroid treatment with doses of at least 10 mg/day of a prednisone equivalent for more than 2 weeks); liver cirrhosis; cancer; smoking (current or former); biochemical parameters; and organ failure at VA-ECMO cannulation.

After VA-ECMO cannulation, we collected data on: reason for initiation of VA-ECMO treatment; VA-ECMO flow (L/min); maximal dose of norepinephrine (μg/kg/min); maximal dose of dobutamine (μg/kg/min); and presence or absence of an intra-aortic balloon pump.

In patients undergoing levosimendan treatment, echocardiographic measurements of aortic velocity–time integral and left ventricular ejection fraction were recorded just before levosimendan initiation and at the end of levosimendan infusion. Echocardiographic measurements were recorded with a VA-ECMO flow of 1 L/min and after interrupting intra-aortic balloon pump.

### Clinical definitions and study endpoints

VA-ECMO weaning failure was defined as death during VA-ECMO treatment or as death within 24 h after VA-ECMO removal [10].

The primary endpoint was the impact of exposure to levosimendan on VA-ECMO weaning.

The secondary endpoint was the impact of exposure to levosimendan on mortality 30 days after VA-ECMO cannulation.

### Statistical analysis

Results were expressed as frequencies and percentages for categorical variables, and as means and standard deviations for continuous variables. Prior to propensity score matching, continuous variables were compared using Student’s *t* test or the Mann–Whitney test, as appropriate. Qualitative variables were compared using Pearson’s Chi-square or Fisher’s exact test, as appropriate. Survival 30 days after VA-ECMO cannulation was estimated using the Kaplan–Meier method and compared using the log-rank test.

The propensity score was defined as the probability of exposure to levosimendan. In order to limit over-adjustment due to the use of this score [14], we selected only the covariates most likely to introduce a confounding bias [15]. The propensity score was estimated using a logistic regression adjusted for age, sex, indication for VA-ECMO, VA duration, presence of a history of heart failure, body mass index > 30 kg/m^2^, Glasgow Coma Scale score on admission and presence of high blood pressure. Matching was then performed between one patient exposed to levosimendan and up to two unexposed patients [16], with a propensity score caliper of 0.05.

After propensity score matching, standardized differences were estimated to compare baseline characteristics and to therefore assess the accuracy of the matching procedure. Associations between outcomes and covariates were assessed using bivariate conditional Cox models stratified by the risk set defined with the propensity score matching procedure. Hazard ratios (HR) and their 95% confidence intervals were calculated.

A two-tailed *p* value < 0.05 was considered significant. Analyses were performed using SAS statistical software (9.4, SAS Institute, Cary, NC, USA).

## Results

### Study population

Over the study period, 201 patients underwent VA-ECMO treatment. Among these, 51 patients were excluded from the study (7 were < 18 years, 20 received VA-ECMO for < 2 days, and 24 were treated with central VA-ECMO). A total of 150 patients supported using peripheral VA-ECMO were eligible for the study.

In 2010–2011, in 2012–2013, in 2014–2015 and in 2016–2017, two out of 10 patients (20%), 17 out of 52 (32.7%), 23 out 63 (36.5%) and 9 out 25 (36%) were, respectively, treated with levosimendan.

**Fig. 1:**
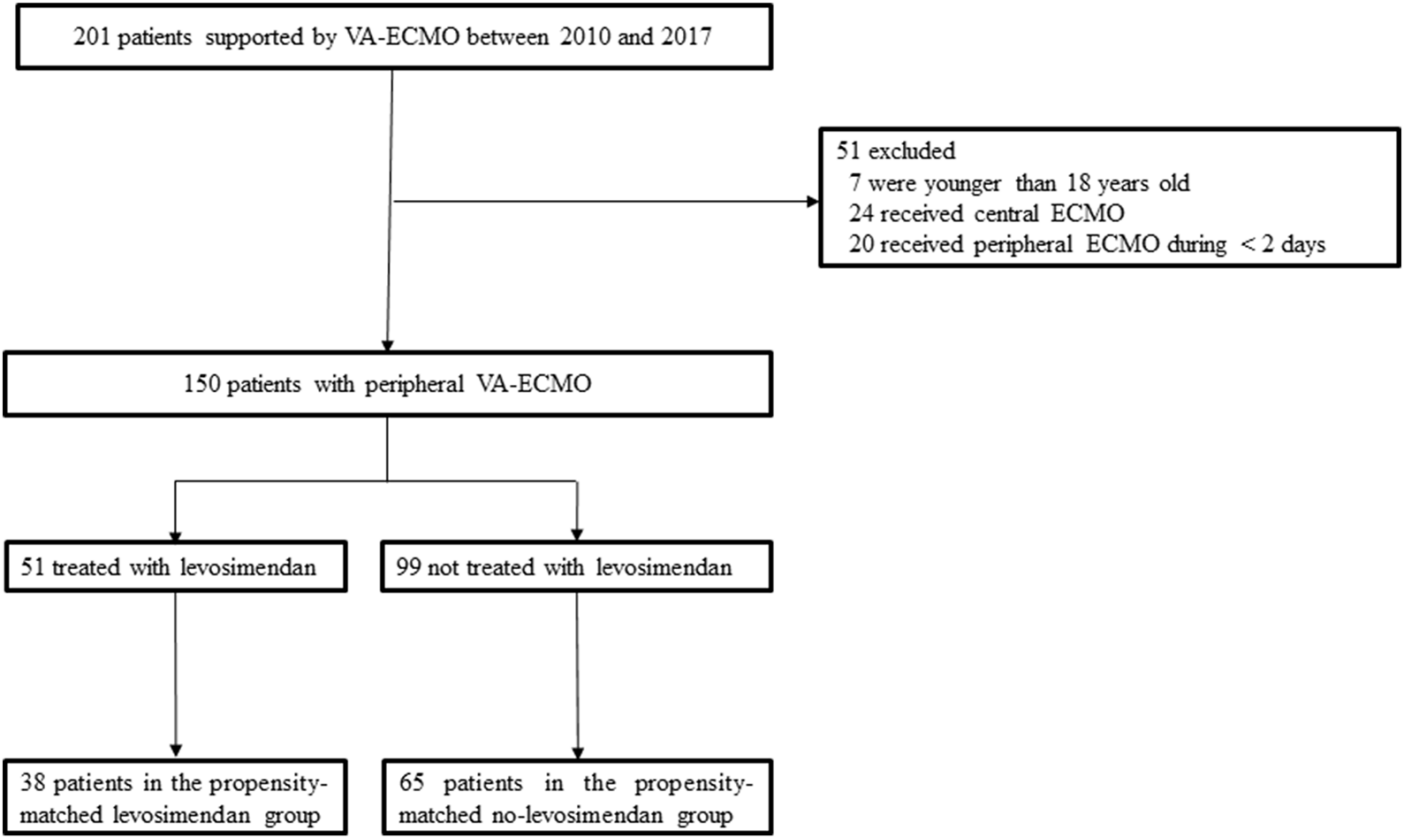
Selection of the study sample

Thirty-eight propensity-matched patients were evaluated in the levosimendan group and 65 in the non-levosimendan group (Fig. 1).

### Characteristics and outcome of the 150 pre-matched patients

Table 1 presents the characteristics of the 150 patients on ICU admission and on study inclusion (prior to matching). Mean age was 53.4 ± 15 years, and median Simplified Acute Physiology Score 2 on admission was 59.2 ± 19.7 (Table 1). During the study period, 51 patients were treated with levosimendan (34%). Main indications for initiation of VA-ECMO treatment were post-cardiotomy cardiogenic shock in 49 cases (32.7%) and post-acute myocardial infarction cardiogenic shock in 44 cases (29.3%). VA-ECMO cannulation site was femoro-femoral in 147 cases (97%), and an intra-aortic balloon pump was present in 42 cases (28%).

**Table 1:** Baseline patient characteristics in pre-matched groups

Variables	Total	Levosimendan		*P* value
	(*n* = 150)	Yes (*n* = 51)	No (*n* = 99)	
Length of stay in hospital before VA-ECMO (days)	5.1 ± 8.6	6.4 ± 8.5	4.4 ± 8.5	0.18
Length of stay in ICU before VA-ECMO (days)	1.1 ± 4.1	0.6 ± 1.6	1.3 ± 4.9	0.16
Clinical characteristics at ICU admission				
Simplified Acute Physiology Score 2	59.2 ± 19.7	55.5 ± 19.6	61 ± 19.5	0.1
Male	98 (65.3)	36 (70.6)	62 (62.6)	0.37
Age (years)	53.4 ± 15	53.6 ± 15.4	53.2 ± 14.9	0.87
Body mass index > 30 kg/m^2^	33 (22)	8 (15.7)	25 (25.3)	0.22
Previous coronary artery disease	44 (29.3)	17 (33.3)	27 (27.3)	0.45
Hypertension	65 (43.3)	20 (39.2)	45 (45.5)	0.49
Chronic renal failure with dialysis	15 (10)	6 (11.8)	9 (9.1)	0.78
Chronic obstructive pulmonary disease	8 (5.3)	3 (5.9)	5 (5.1)	1
Diabetes mellitus	54 (36)	19 (37.3)	35 (35.4)	0.86
History of congestive heart failure	35 (23.3)	17 (33.3)	18 (18.2)	0.04
Immunosuppression	5 (3.3)	2 (3.9)	3 (3)	1
Liver cirrhosis	3 (2)	2 (3.9)	1 (1)	0.27
Cancer	1 (0.7)	0	1 (1)	1
Smoking (current or former)	47 (31.3)	17 (33.3)	30 (30.3)	0.71
Hazardous alcohol use	30 (20)	11 (21.6)	19 (19.2)	0.83
Glasgow Coma Scale score	12.7 ± 4.6	13.8 ± 3.6	12.2 ± 4.9	0.029
Catecholamines	149 (99.3)	51 (100)	98 (99)	1
Mechanical ventilation	137 (91.3)	47 (92.2)	90 (90.9)	1
Renal replacement therapy	61 (40.7)	20 (39.2)	41 (41.4)	0.86
Total bilirubin level (µmol/L)	31.6 ± 39.8	35.5 ± 50.8	29.6 ± 32.9	0.49
Platelet count (G/L)	154 ± 93	160 ± 107	151 ± 85	0.56
Prothrombin (%)	47.5 ± 20	50.3 ± 16.8	46.1 ± 21.4	0.22
Hemoglobin level (g/dL)	10.3 ± 7.5	11.5 ± 12.5	9.6 ± 2.3	0.16
Creatinine level (µmol/L)	172 ± 138	173 ± 131	171 ± 143	0.45
Left ventricular ejection fraction (%)	19.9 ± 6.7	19.1 ± 6.8	20.3 ± 6.6	0.36
Reason for VA-ECMO				0.024
Cardiac arrest	4 (2.7)	2 (3.9)	2 (2)	
Dilated cardiomyopathy	10 (6.7)	5 (9.8)	5 (5.1)	
Myocarditis	7 (4.7)	1 (2)	6 (6.1)	
Acute myocardial infarction	44 (29.3)	14 (27.5)	30 (30.3)	
Post-cardiotomy	49 (32.7)	24 (47.1)	25 (25.3)	
Acute respiratory distress syndrome	3 (2)	0	3 (3)	
Percutaneous implantation of VA-ECMO	66 (44)	21 (41.2)	45 (45.5)	0.73
Hemodynamic parameters (first 24 h post-VA-ECMO)				
VA-ECMO flow (L/min)	3.65 ± 0.62	3.87 ± 0.69	3.59 ± 0.61	0.29
Maximal dose of norepinephrine (µg/kg/min)	0.74 ± 0.69	0.74 ± 0.62	0.74 ± 0.72	0.62
Maximal dose of Dobutamine (µg/kg/min)	10.1 ± 4.3	10.7 ± 4.5	9.8 ± 4.2	0.32
Intra-aortic balloon pump	42 (28)	16 (31.4)	26 (26.3)	0.32
VA-ECMO duration (day)	11.6 ± 11	12.3 ± 11.8	11.2 ± 10.6	0.23

Results are expressed as mean ± SD or number (%)

*VA-ECMO* venoarterial extracorporeal membrane oxygenation, *ICU* intensive care unit

The proportion of patients with a history of congestive heart failure (33.3% vs. 18.2%, *P* = 0.04) was higher in the levosimendan group than in the non-levosimendan group. Reasons for VA-ECMO initiation varied between the two groups (*P* = 0.024) (Table 1).

Levosimendan was administered after 3.2 ± 2.8 days after VA-ECMO cannulation. In patients treated with levosimendan, left ventricular ejection fraction increased from 21.5 ± 9.1% to 30.7 ± 13.5% (*P* < 0.0001) and aortic velocity–time integral increased from 8.9 ± 4 cm to 12.5 ± 3.8 cm, (*P* = 0.002). Out of 150 patients, 103 were weaned from VA-ECMO (68.7%): 42 (82.4%) in the levosimendan group versus 61 (61.6%) in the non-levosimendan group (*P* = 0.01).

**Fig. 2:**
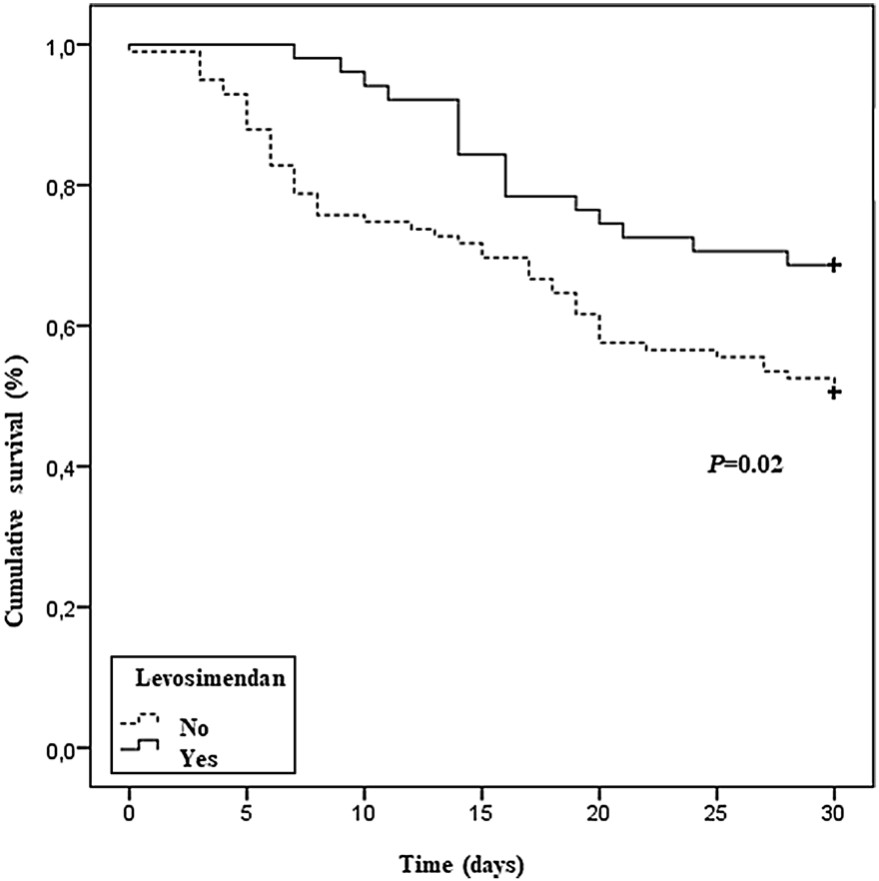
Survival rate for patients with or without levosimendan by Kaplan–Meier analysis

Kaplan–Meier survival curves showed that survival rate at 30 days was 78.4% in the levosimendan group and 49.5% in the non-levosimendan group (*P* = 0.02) (Fig. 2).

### Characteristics and outcome of the 103 matched patients

After propensity score matching, no significant differences were found in the characteristics of patients between the levosimendan group and the non-levosimendan group (Table 2).

**Table 2:** Patient characteristics in propensity-matched groups

Variables	Levosimendan group		Standardized difference
	Yes (*n* = 38)	No (*n* = 65)	
Length of stay in hospital before VA-ECMO (days)	4.7 ± 6.7	5.4 ± 9.7	− 0.09
Length of stay in ICU before VA-ECMO (days)	0.5 ± 1.8	1.7 ± 5.5	− 0.29
Clinical characteristics at ICU admission			
Simplified Acute Physiology Score 2	58.3 ± 18.1	58.1 ± 19.4	0.01
Male	26 (68.4)	46 (70.8)	− 0.05
Age (years)	53.8 ± 15.4	54.2 ± 14.5	− 0.03
Body mass index > 30 kg/m^2^	8 (21.1)	13 (20.0)	0.03
Previous coronary artery disease	17 (44.7)	17 (26.2)	0.39
Hypertension	15 (39.5)	29 (44.6)	− 0.10
Chronic renal failure with dialysis	6 (15.8)	8 (12.3)	0.10
Chronic obstructive pulmonary disease	3 (7.9)	5 (7.7)	0.01
Diabetes mellitus	17 (44.7)	21 (32.3)	0.25
History of congestive heart failure	9 (23.7)	13 (20.0)	0.09
Immunosuppression	1 (2.6)	1 (1.5)	0.08
Liver cirrhosis	1 (2.6)	1 (1.5)	0.08
Cancer	0	0	0.00
Smoking (current or former)	12 (31.6)	24 (36.9)	− 0.11
Hazardous alcohol use	8 (21.1)	13 (20.0)	0.03
Glasgow Coma Scale score	13.7 ± 3.7	13.4 ± 3.9	0.08
Catecholamines	38 (100.0)	64 (98.5)	0.18
Mechanical ventilation	36 (94.7)	57 (87.7)	0.25
Renal replacement therapy	17 (44.7)	29 (44.6)	0.00
Total bilirubin level (µmol/L)	35.1 ± 50.6	32.7 ± 37.3	0.05
Platelet count (G/L)	169.4 ± 116.0	144.5 ± 89.9	0.24
Prothrombin (%)	51.1 ± 16.1	45.4 ± 22.3	0.29
Hemoglobin level (g/dL)	12.2 ± 14.4	9.7 ± 2.4	0.25
Creatinine level (µmol/L)	182.0 ± 149.5	181.7 ± 165.2	0.00
Left ventricular ejection fraction (%)	19.6 ± 6.8	20.6 ± 6.8	− 0.14
Reason for VA-ECMO			0.24
Cardiac arrest	1 (2.6)	2 (3.1)	
Dilated cardiomyopathy	4 (10.5)	5 (7.7)	
Myocarditis	1 (2.6)	4 (6.1)	
Acute myocardial infarction	13 (34.2)	18 (27.7)	
Post-cardiotomy	14 (36.9)	24 (36.9)	
Other reason	5 (13.2)	12 (18.5)	
Percutaneous implantation of VA-ECMO	18 (47.4)	27 (41.5)	0.12
Hemodynamic parameters (first 24 h post-VA-ECMO)			
VA-ECMO flow (L/min)	3.7 ± 0.6	3.5 ± 0.6	0.25
Maximal dose of norepinephrine (µg/kg/min)	0.7 ± 0.6	0.7 ± 0.6	0.04
Maximal dose of Dobutamine (µg/kg/min)	10.8 ± 4.3	9.9 ± 3.6	0.23
Intra-aortic balloon pump	15 (39.5)	17 (26.2)	0.28
VA-ECMO duration	11.9 ± 8.1	10.7 ± 8	0.15

Results are expressed as mean ± SD or number (%)

*VA-ECMO* venoarterial extracorporeal membrane oxygenation, *ICU* intensive care unit

Out of 103 patients, 73 were successfully weaned from VA-ECMO (70.9%). After propensity score matching, exposure to levosimendan was the only remaining factor associated with a significant reduction in VA-ECMO weaning failure rates (hazard ratio = 0.16; 95% confidence interval: 0.04–0.70; *P* = 0.01) (Table 3).

**Table 3:** Factors associated with VA-ECMO weaning after propensity score matching

Variables	Success (*n* = 73)	Failure (*n* = 30)	*P*-value
Length of stay in hospital before VA-ECMO (day)	5.6 ± 8.5	3.9 ± 9.1	0.35
Length of stay in ICU before VA-ECMO (day)	1.4 ± 5.1	0.7 ± 1.1	0.19
Clinical characteristics at ICU admission			
Simplified Acute Physiology Score 2	56.6 ± 19.0	62.1 ± 18.2	0.18
Male	50 (68.5)	22 (73.3)	0.63
Age (years)	52.5 ± 15.8	57.9 ± 11.1	0.09
Body mass index > 30 kg/m^2^	13 (17.8)	8 (26.7)	0.31
Previous coronary artery disease	22 (30.1)	12 (40.0)	0.33
Hypertension	27 (37.0)	17 (56.7)	0.07
Chronic renal failure with dialysis	10 (13.7)	4 (13.3)	1.00
Chronic obstructive pulmonary disease	4 (5.5)	4 (13.3)	0.23
Diabetes mellitus	23 (31.5)	15 (50.0)	0.08
History of congestive heart failure	18 (24.7)	4 (13.3)	0.2
Immunosuppression	2 (2.7)	0 (0.0)	1.0
Liver cirrhosis	2 (2.7)	0 (0.0)	1.00
Cancer	0	0	1
Smoking (current or former)	24 (32.9)	12 (40.0)	0.49
Hazardous alcohol use	14 (19.2)	7 (23.3)	0.63
Glasgow Coma Scale score	13.6 ± 3.8	13.3 ± 4.1	0.73
Catecholamines	72 (98.6)	30 (100.0)	1.00
Mechanical ventilation	65 (89.0)	28 (93.3)	0.72
Renal replacement therapy	31 (42.5)	15 (50.0)	0.48
Total bilirubin level (µmol/L)	35.9 ± 44.2	28.0 ± 37.8	0.39
Platelet count (G/L)	152.3 ± 102.8	157.1 ± 96.2	0.82
Prothrombin (%)	49.3 ± 21.4	43.3 ± 17.2	0.18
Hemoglobin level (g/dL)	10.9 ± 10.6	9.9 ± 2.1	0.59
Creatinine level (µmol/L)	173.8 ± 138.4	201.4 ± 201.5	0.43
Left ventricular ejection fraction (%)	20.2 ± 6.5	20.3 ± 7.4	0.97
Reason for VA-ECMO			0.28
Cardiac arrest	1 (1.4)	2 (6.7)	
Dilated cardiomyopathy	8 (11.0)	1 (3.3)	
Myocarditis	5 (6.8)	0 (0.0)	
Acute myocardial infarction	20 (27.4)	11 (36.7)	
Post-cardiotomy	28 (38.4)	10 (33.3)	
Other reason	11 (15.1)	6 (20.0)	
Percutaneous implantation of VA-ECMO	34 (46.6)	11 (36.7)	0.36
Hemodynamic parameters (first 24 h post-VA-ECMO)			
VA-ECMO flow (L/min)	3.5 ± 0.6	3.6 ± 0.5	0.51
Maximal dose of norepinephrine (µg/kg/min)	0.8 ± 0.6	0.6 ± 0.6	0.15
Maximal dose of dobutamine (µg/kg/min)	10.1 ± 3.7	10.4 ± 4.5	0.69
Intra-aortic balloon pump	24 (32.9)	8 (26.7)	0.54
VA-ECMO duration	11.9 ± 8.8	9.3 ± 5.4	0.14
Levosimendan	32 (43.8)	6 (20.0)	0.01

Results are expressed as mean ± SD or number (%)

*VA-ECMO* venoarterial extracorporeal membrane oxygenation, *ICU* intensive care unit

The use of levosimendan tended to decrease 30-day mortality after propensity score matching; however, the difference in 30-day mortality between the two groups was not significant (hazard ratio = 0.55; 95% confidence interval: 0.27–1.10; *P* = 0.09). The other factors found to be associated with 30-day mortality were higher Simplified Acute Physiology Score 2 (*P* = 0.002), higher age (*P* = 0.01) and reason for VA-ECMO (*P* = 0.01).

## Discussion

This retrospective study suggests that levosimendan might be associated with beneficial effects on peripheral VA-ECMO weaning in patients hospitalized in ICU. The decrease in mortality did not reach statistical significance (*P* = 0.09) among propensity-matched patients.

To our knowledge, two other studies have evaluated the impact of levosimendan on VA-ECMO weaning [10, 11]. This first study, conducted by Distelmaier et al. [9], also found a beneficial effect of levosimendan, but it was restricted to patients treated with the drug after cardiac surgery [9].

The second retrospective study by Jacky et al. was also restricted to cardiac surgery patients and compared levosimendan to milrinone without finding any significant difference between the two treatments [11]. By contrast, all patients undergoing peripheral VA-ECMO were included in our analysis, including those who did not undergo cardiac surgery. In our view, extending the study population to non-cardiac surgery patients is relevant for physicians in the field because cardiac surgery is not the only indication for VA-ECMO. Indeed, in both our study and the Extracorporeal Life Support Organization Registry, over 50% of patients had medical indication for VA-ECMO [17].

Studies have shown that the incidence of severe complications like cannula-related infection [18], severe bleeding [19] and thromboembolic event [20] is associated with longer VA-ECMO duration. The use of levosimendan in patients undergoing VA-ECMO may therefore be of interest both to reduce the duration of mechanical support and to minimize severe complications.

In addition to its hemodynamic actions, another hypothesis for the beneficial effects of levosimendan may be its positive protective effects on endothelium function, particularly in inflammatory situations [21, 22]. This is relevant to VA-ECMO treatment [8], which has been shown to be associated with endothelial damage and pro-inflammatory effects [23, 24]. Thus, in a preliminary observational study, Sangalli et al. [8] found that infusion of levosimendan leads to significant improvement in endothelial function, cardiac index and mixed venous oxygen saturation in adult cardiogenic shock patients with low ejection supported using VA-ECMO. They concluded that levosimendan facilitates weaning from extracorporeal support.

In our study, a nonsignificant decrease in mortality was found after propensity score matching. However, this lack of significance may be due to a lack of power, as only 103 patients were ultimately evaluated. By contrast, the study by Distelmaier et al. found an association between levosimendan treatment and improved survival in patients undergoing VA-ECMO after cardiac surgery [10].

A large study evaluating 5263 patients treated with VA-ECMO found that 64.4% of these patients were successfully weaned from extracorporeal support. However, VA-ECMO weaning did not necessarily result in survival, as in-hospital mortality in weaned patients was over 38% [25].

The beneficial effects of levosimendan are still debated in clinical practice [5–7]. In two meta-analyses of randomized studies evaluating patients after cardiac surgery, levosimendan was shown to have a greater effect on mortality in patients with impaired left ventricular systolic function than in those with preserved left ventricular systolic function [26, 27]. Moreover, randomized controlled trials found that the use of levosimendan was not associated with beneficial effects on duration of mechanical ventilation, ICU length of stay or mortality [6, 28]. It may be that levosimendan should be administered only to specific cardiogenic shock patients, in particular those undergoing VA-ECMO. Levosimendan may be associated with favorable outcome—including reduced mortality—in patients with very low left ventricular ejection fraction.

## Limitations

Our study has several limitations. The retrospective nature of the analysis is clearly a weakness. In addition, our study may suffer from a lack of power because of the low number of patients and events evaluated. The main limitation of our study is that administration of levosimendan was not randomized. However, to limit biases due to the absence of randomization, we used a multivariate logistic regression model with a propensity score analysis. Although several definitions of VA-ECMO weaning success have been proposed [11, 29, 30], we adopted the definition used in the recent study by Distelmeier et al. [10]. Aortic velocity–time integral and left ventricular ejection fraction were not collected in the control group. And it is possible that 24 h of mechanical support provide some improvement in aortic velocity–time integral even in the absence of levosimendan. The timing of levosimendan administration with respect to VA-ECMO initiation was variable, and so, the time, the conditions and the optimum conditions for introducing this treatment in this context remain to be established. As in the study Distelmaier et al. [10], the preparation used for levosimendan (0.125 mg/mL) was not the one recommended for levosimendan administration (< 0.05 mg/mL) [31] and that may have resulted in potential inhomogeneity of the drug delivery with medication precipitation.

## Conclusion

This study suggests that levosimendan might be associated with a beneficial effect on VA-ECMO weaning in ICU patients. The difference in mortality among propensity-matched patients failed to reach statistical significance.

## Abbreviations

VA-ECMO: venoarterial extracorporeal membrane oxygenation
ICU: intensive care unit
